# Use of mNUTRIC-Score for Nutrition Risk Assessment and Prognosis Prediction in Critically Ill Patients with COVID-19: A Retrospective Observational Study

**DOI:** 10.1155/2021/5866468

**Published:** 2021-12-22

**Authors:** Francisco G. Yanowsky-Escatell, Areli L. Ontiveros-Galindo, Kevin J. Arellano-Arteaga, Luis M. Román-Pintos, Carlos A. Andrade-Castellanos, Diana M. Hernández-Corona, Tonatiuh González-Heredia, Geannyne Villegas-Rivera

**Affiliations:** ^1^Departamento de Medicina Interna, Hospital Civil de Guadalajara Dr. Juan I. Menchaca, Guadalajara, Jalisco, Mexico; ^2^Programa de Doctorado en Investigación Multidisciplinaria en Salud, Centro Universitario de Tonalá, Universidad de Guadalajara, Guadalajara, Jalisco, Mexico; ^3^Departamento de Ciencias de la Salud-Enfermedad como Proceso Individual, Centro Universitario de Tonalá, Universidad de Guadalajara, Guadalajara, Jalisco, Mexico; ^4^Departamento de Ciencias Biomédicas, Centro Universitario de Tonalá, Universidad de Guadalajara, Guadalajara, Jalisco, Mexico

## Abstract

**Introduction:**

Nutritional risk is highly prevalent in patients with COVID-19. Relevant data on nutritional assessment in the critically ill population are scarce. This study was conducted to evaluate the modified Nutrition Risk in the Critically Ill (mNUTRIC)-Score as a mortality risk factor in mechanically ventilated patients with COVID-19.

**Methods:**

We conducted this retrospective observational study in critically ill patients with COVID-19. Patients' characteristics and clinical information were obtained from electronic medical records. The nutritional risk for each patient was assessed at the time of mechanical ventilation using the mNUTRIC-Score. The major outcome was 28-day mortality.

**Results:**

Ninety-eight patients were analyzed (mean age, 57.22 ± 13.66 years, 68.4% male); 46.9% of critically ill COVID-19 patients were categorized as being at high nutrition risk (mNUTRIC-Score of ≥5). A multivariate logistic regression model indicated that high nutritional risk has higher 28-day hospital mortality (OR = 4.206, 95% CI: 1.147–15.425, *p*=0.030). A multivariate Cox regression analysis showed that high-risk mNUTRIC-Score had a significantly increased full-length mortality risk during hospitalization (OR = 1.991, 95% CI: 1.219–3.252, *p*=0.006).

**Conclusion:**

The mNUTRIC-Score is an independent mortality risk factor during hospitalization in critically ill COVID-19 patients.

## 1. Introduction

The severe acute respiratory syndrome coronavirus 2 (SARS-CoV-2), the causative agent of coronavirus disease 2019 (COVID-19), has attacked Latin American countries with varying intensity. Mexico in particular has some of the highest number of confirmed cases and COVID-related deaths in the world [1].

As COVID-19 cases and deaths continue to increase in our region, the role of nutritional assessment in these patients is gradually gaining momentum [2–5]. Malnutrition and risk of malnutrition has been associated with increased mortality and poor long-term outcomes in certain populations [6, 7]. This is particularly true in patients with pneumonia in which impaired muscle and respiratory function is invariably present [8].

SARS-CoV-2 infection is associated with a catabolic state and anabolic resistance leading to increased nutritional demand. Dietary intake reduction in COVID-19 population is fairly common. Studies have described anorexia, dysgeusia, and anosmia as important factors leading to fasting and weight loss [9]. Therefore, the identification of the risk and presence of malnutrition is considered an integral part of the approach to patients with COVID-19 in general healthcare [10].

The Nutrition Risk in the Critically Ill (NUTRIC)-Score is calculated retrospectively including age, routinely severity scores, comorbidity numbers, and pre-ICU hospital length of stay to identify patients who will benefit from early nutrition therapy [11, 12]. In its original version, interleukin-6 (IL-6) was used as an inflammatory marker associated with nutritional risk. However, due to the difficulty of obtaining this measurement in regular clinical practice, the score was later validated without the use of IL-6 [12–14]. The adjusted score was called the modified NUTRIC (mNUTRIC)-Score. During the COVID-19 pandemic, an increased prevalence of high nutritional risk has been reported in other studies of critically ill patients using the mNUTRIC-Score [15–17].

The aim of the present study was to evaluate mNUTRIC-Score as a mortality risk factor in mechanically ventilated patients with COVID-19 and, also, to analyze nutritional risk and clinical outcomes in mechanically ventilated patients with COVID-19 in a tertiary care hospital in Mexico.

## 2. Patients and Methods

**Figure 1:**
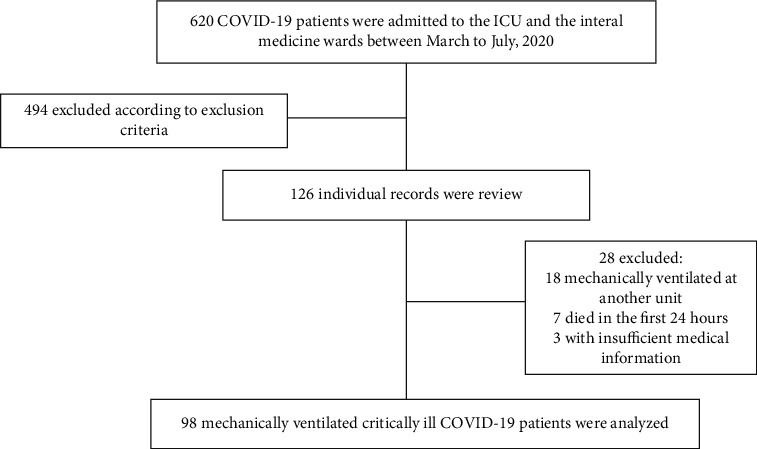
Flow diagram of the study.

This is a retrospective observational cohort study conducted at the Hospital Civil de Guadalajara Dr. Juan I. Menchaca, a tertiary care hospital in Jalisco, Mexico. All patients admitted to the adult ICU and the internal medicine wards diagnosed with COVID-19, defined by a positive reverse-transcriptase-polymerase-chain-reaction (RT-PCR) test for SARS-CoV-2 by nasopharyngeal swab, from March to July 2020, and for whom the necessary information was available, were included in the study. Patients under the age of 18 years, pregnant patients, patient requiring surgical intervention, and patients not requiring mechanical ventilation were excluded from the study. The final cohort included 98 individual records (Figure 1), and the project was approved by the Institutional Review Board under research protocol no. 04553/20, HCJIM/2021, and permitted by our hospital Ethics Committee.

### 2.1. Clinical Variables and Data Collection

We reviewed data related to demographics, clinical characteristics, and comorbidities (diabetes, hypertension, coronary heart disease, chronic kidney disease, and chronic obstructive pulmonary disease) from electronic health records through a standardized form. We collected the following variables: hospital length of stay, length of mechanical ventilation, and 28-day mortality. The partial pressure of arterial oxygen to the fraction of inspired oxygen (PaO_2_/FiO_2_) ratio, Sequential Organ Failure Assessment (SOFA) score, the Acute Physiology and Chronic Health Evaluation II (APACHE II), and laboratory values that included glucose, creatinine, blood urea nitrogen (BUN), albumin, inflammatory markers (C-reactive protein), electrolytes, and acid-base status were collected at the time of mechanical ventilation. The nutritional risk for each patient was assessed on the day of mechanical ventilation initiation using the mNUTRIC-Score (0 to 9 points). This score was calculated based on the NUTRIC-Score by eliminating IL-6 values. A score of ≥5 indicated a high nutritional risk [12, 13]. Patients who received nutrition therapy within 48 h after the initiation of mechanical ventilation were considered early feeding cases according to the European Society for Clinical Nutrition and Metabolism (ESPEN) guidelines and >48 h were considered late feeding [18].

### 2.2. Statistical Analysis

All statistical analyzes were performed with SPSS version 21.0 (SPSS Inc., Chicago, IL, USA). A *p* value <0.05 was considered statistically significant. Descriptive statistics were displayed as frequencies and percentages for categorical variables and means and standard deviation (SD) or median (min-max) for quantitative variables, depending on the distribution of variables (nonparametric or parametric) assessed by the Kolmogorov–Smirnov test. The chi-square test was used for categorical variables; continuous variables were compared and analyzed using the paired-samples *t*-test or the Mann–Whitney *U* test. Logistic regression was used to analyze the association between clinical and nutritional factors with a risk of inpatient death. Then, we performed a multivariate Cox regression to establish the relationships between the mNUTRIC-Score and mortality in critically ill patients with COVID-19.

## 3. Results

### 3.1. Characteristics of the Study Population

A total of 98 patients with laboratory-confirmed COVID-19 were included in this study. Their demographic and clinical characteristics are listed in Table 1. The mean age of the study cohort was 57.22 ± 13.66 years, and the majority of our patients were male (68.4%). Hypertension (44.9%), diabetes (35.7%), coronary heart disease (17.3%), and chronic kidney disease (8.2%) were the four most common comorbidities. The median length of stay was 13 days (2–96 days), and the median length of mechanical ventilation duration was 7.5 days (1–40 days). Patients developed several complications, including acute kidney injury (63.3%) and upper gastrointestinal bleeding (6.1%); the proportion of patients who were treated with vasopressors was considerable (51%). At the time of mechanical ventilation, patients had a mean SOFA score of 7.69 ± 2.69 and a mean APACHE II score of 19.06 ± 6.92. The 28-day in-hospital mortality rate was 72.4%, and those who died during the whole hospitalization were 77.6% (Table 1).

The median time to initiate feeding was 74 hours. Most patients received enteral nutrition (EN) (77.6%), none received parenteral nutrition (PN), and 22.4% did not receive any nutritional support as a result of contraindications (Table 1).

### 3.2. Nutritional Risk and Outcomes

Patients were divided into high nutritional risk if they had a mNUTRIC-Score ≥5 and low nutritional risk if they had a mNUTRIC-Score <5. Of the 98 patients available for the evaluation of the mNUTRIC-Score, 46.9% of the patients were classified as high nutritional risk and 53.1% of the patients as low nutritional risk. The median mNUTRIC-Score in the high-risk group was 6 (5–8). These groups of patients suffered greater comorbidities (evaluated with the Charlson comorbidity index) and greater incidence of acute kidney injury (73.9% vs. 53.8%). There were no differences in the incidence of upper gastrointestinal bleeding or in terms of use of vasopressors between the groups. Significant differences concerning kidney function were observed between high- and low-risk groups: creatinine was 2.04 mg/dL vs. 0.96 mg/dL (*p*=≤0.001) and BUN was 47.40 vs. 27.50 (*p*=≤0.001), respectively. C-reactive protein was significantly higher in patients with mNUTRIC-Score ≥5 (230.28 ± 150.89 vs. 171.77 ± 116.6; *p*=0.033). Mortality at 28-days was 91.3% in the high-nutritional-risk group and 55.8% in the low-nutritional-risk group (Table 2).

Eighteen patients (39.1%) in the high-nutritional-risk group received early feeding versus seventeen (32.6%) in the low-nutritional-risk group (*p*=0.193). The 28-day mortality rate was not different between these 2 groups (*p*=0.490). SOFA, APACHE II, and Charlson comorbidity index at admission were significantly higher in deaths than in survivors (Table 3). Elevated levels of blood urea nitrogen and creatinine were also observed in deaths, compared to survivors (*p* ≤ 0.001).

**Figure 2:**
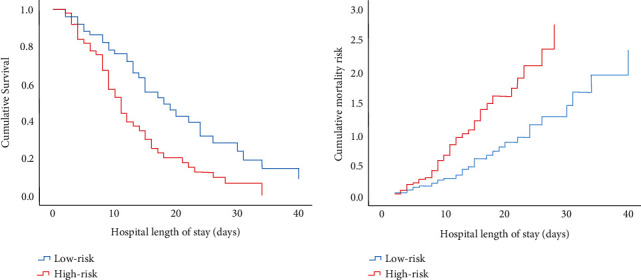
Cumulative survival and mortality risk multivariate Cox regression analysis.

Logistic regression was performed for those variables, and we found age, Charlson comorbidity index, SOFA, APACHE II, BUN, mNUTRIC-Score, and acute kidney injury as 28-day mortality risk factors (Table 4). We divided patients into high- and low-risk mNUTRIC-Scores and found a strong association of ≥5 points with the risk of death in the hospital (OR = 8.328, 95% CI: 2.604–26.630, *p*=≤0.001). After adjusting for gender, age, and Charlson comorbidity index, a high-risk mNUTRIC-Score was an independent 28-day mortality risk factor (OR = 4.206, 95% CI: 1.147–15.425, *p*=0.030). The multivariate Cox regression analysis showed that after adjusting for age, Charlson comorbidity index, and acute kidney injury, patients with high-risk mNUTRIC-Score had a significantly increased full-length mortality risk during hospitalization (OR = 1.991, 95% CI: 1.219–3.252, *p*=0.006) (Figure 2).

## 4. Discussion

This study demonstrates that the majority of critically ill patients with COVID-19 admitted to both the ICU and the internal medicine wards had a high nutritional risk. The significance of evaluating nutritional risk has been gathering clinical attention for its usefulness as a prognostic indicator in COVID-19 patients. Zhang et al. [15], in a retrospective study conducted in China, reported that a large proportion of critically ill COVID-19 patients had a high nutritional risk (61%) as revealed by their mNUTRIC-Score. High-nutritional-risk scores were similarly prevalent (66%) in an observational study conducted in the central region of Mexico [17]. In both studies, the mNUTRIC-Score was able to independently predict the risk of death at 28 days. Similar findings were observed in a larger study conducted by Li et al. [16], showing a significantly higher mortality in the high-nutritional-risk group (mNUTRIC-Score ≥5 points). The results of the present study could not be fully compared with those of previous studies, as only 46.9% of our patients had a high nutritional risk. Nevertheless, a mNUTRIC-Score ≥5 was consistently demonstrated to be associated with increased mortality risk in our study.

According to the mNUTRIC-Score, up to 40% of mechanically ventilated patients without COVID-19 are at nutritional risk [19–21], with higher scores directly proportional to the average length of stay in the ICU and mortality. Recent evidence indicates that COVID-19 patients are at even higher risk of malnutrition and mortality as demonstrated by mNUTRIC-Scores [15–17, 22], supporting the concept that SARS-CoV-2-mediated inflammatory state contributes to malnutrition and a poor prognosis. This could be explained as follows: First, muscle protein is consumed by the acute inflammatory response of SARS-CoV-2 infection. Cytokine storm of interferon-*α*, interferon-*γ*, C-reactive protein, IL-6, IL-12, and tumor necrosis factor-*α* leads to metabolic stress and muscle catabolism (skeletal muscle is catabolized to provide the immune system with amino acids). Second, angiotensin-converting enzyme 2 is highly expressed in the gastrointestinal track [23]. Gastrointestinal symptoms such as diarrhea, abdominal pain, nausea, and vomiting are fairly common, accelerating the occurrence of malnutrition in patients with COVID-19. Poor appetite is related to anxiety (fear of their illness, isolation treatment, lack of normal social communication, etc.) which, in addition to anosmia and dysgeusia, could further aggravate malnutrition [9, 24].

The limited use of PN observed during the present study is in line with nutritional practices in critically ill adult ICU patients in Latin America, where less than one in ten patients received PN alone [25]. PN should be considered in the event of contraindication for EN, if the objectives cannot be achieved with EN alone or in patients with gastrointestinal intolerance [18], a common occurrence in patients with COVID-19 admitted to the ICU [26].

Early EN, started within 48 h after ICU admission, preserves the intestinal mucosal barrier and has a beneficial effect on the reduction of enterogenic infections [18, 27]. In our study, we observed a significant gap between the ESPEN recommendations and the actual feeding performance, as only 35.7% of the patients received EN within 48 h. The initiation of EN may receive a lower priority compared to other interventions in critically ill patients with COVID-19 (airway-related procedures, imaging procedures, proning or supination process, physical therapy, nursing care, etc.). Inadequate training and knowledge regarding the principles of clinical nutrition could also impede the provision of nutrition support in a timely manner [28]. We observed no significant difference in mortality between patients who received early and delayed nutrition therapy in our study. This is in accordance with previous research articles and a systemic review from the Cochrane Collaboration [16, 29].

This study has several limitations related to its retrospective design. First, the study was conducted at a single hospital; therefore, the generalizability of the results may be challenged. Second, only 98 critically ill patients with COVID-19 were included in our study (statistical underpower cannot be ruled out). Third, there were no data in the electronic medical records that provide information on caloric and protein provision for the study population. Therefore, the association between nutritional adequacy, mNUTRIC-Scores, and mortality could not be explored.

## 5. Conclusions

Our data suggest that the mNUTRIC-Score, a fast and practical instrument, may be an appropriate tool for nutritional risk assessment and mortality risk for critically ill COVID-19 patients. These findings are important given the ongoing burden of the COVID-19 pandemic. Further prospective studies are needed to support our findings.

## Figures and Tables

**Table 1:** Characteristics of mechanically ventilated critically ill COVID-19 patients.

	*n* = 98
Age, years	57.22 ± 13.66
Male, *n* (%)	67 (68.4%)
Comorbidities	1 (0–7)
Diabetes, *n* (%)	35 (35.7%)
Hypertension, *n* (%)	44 (44.9%)
Coronary heart disease, *n* (%)	17 (17.3%)
Chronic kidney disease, *n* (%)	8 (8.2%)
Chronic obstructive pulmonary disease, *n* (%)	4 (4.1%)
Charlson comorbidity index	2 (0–9)
Severity of disease	
PaO_2_/FiO_2_ ratio	74 (31–366)
SOFA score	7.69 ± 2.69
APACHE II score	19.06 ± 6.92
Nutritional characteristics	
mNUTRIC-Score	4 (0–8)
Enteral nutrition, *n* (%)	76 (77.6%)
No nutritional support, *n* (%)	22 (22.4%)
Initial time of nutrition support	
≤48 h, *n* (%)	35 (35.7%)
>48 h, *n* (%)	41 (41.8%)
Biochemicals	
Glucose (mg/dL)	162.50 (45–533)
Creatinine (mg/dL)	1.25 (0.30–20.23)
BUN (mg/dL)	31.45 (9–137)
C-reactive protein (mg/dL)	199.23 ± 136.30
Sodium (mmol/L)	135.46 ± 5.39
Potassium (mmol/L)	4.45 (2.80–8.90)
Magnesium (mg/dL)	2.29 ± 0.39
Phosphorus (mg/dL)	3.65 (2–12.90)
Lactate (mmol/L)	2 (0.60–7)
Outcomes	
Death at 28-day, *n* (%)	71 (72.4%)

Abbreviations: APACHE II, Acute Physiology and Chronic Health Evaluation II; BUN, blood urea nitrogen; mNUTRIC-Score, modified Nutrition Risk in the Critically Ill-Score; PaO_2_/FiO_2_ ratio, partial pressure of arterial oxygen to fraction of inspired oxygen; SOFA, Sequential Organ Failure Assessment.

**Table 2:** Characteristics and laboratory indices among patients with high and low nutritional risk.

	High-nutritional-risk group (mNUTRIC-Score ≥5, *n* = 46)	Low-nutritional-risk group (mNUTRIC-Score <5, *n* = 52)	*p* value
Clinical characteristics			
Female, *n* (%)	19 (41.3%)	12 (23.1%)	0.053
Male, *n* (%)	27 (58.7%)	40 (76.9%)	
Age, mean	61.91 ± 12.39	53.07 ± 13.49	≤0.001
Comorbidities	2 (0–7)	1 (0–5)	≤0.001
Charlson comorbidity index	4 (2–9)	2 (0–5)	≤0.001
Biochemicals			
Glucose (mg/dL)	175 (88–452)	162 (77–533)	0.105
Creatinine (mg/dL)	2.04 (0.64–20.23)	0.96 (0.39–2.34)	≤0.001
BUN (mg/dL)	47.40 (13–137)	27.50 (9–55)	≤0.001
Albumin (g/dL)	2.47 ± 0.49	2.63 ± 0.46	0.115
C-reactive protein (mg/dL)	230.28 ± 150.89	171.77 ± 116.6	0.033
Arterial blood gas			
pH	7.29 ± 0.14	7.34 ± 0.14	0.114
PaCO_2_ (mmHg)	38 (13–115)	44 (14–163)	0.378
Bicarbonate (mmol/L)	21.09 ± 7.06	24.19 ± 4.93	0.013
Lactate (mmol/L)	2.10 (0.70–7)	2 (0.60–4.9)	0.889
PaO_2_/FiO_2_ (mmHg)	69 (33–366)	84 (31–360)	0.020
Outcomes			
Mechanical ventilation (days)	10 (3–33)	10 (3–40)	0.162
Total mNUTRIC-Score, median (IQR), points	6 (5–8)	2 (0–4)	≤0.001
Hospital length of stay (days)	10.5 (2–34)	15 (2–96)	0.004
Death at 28th day, *n* (%)	42 (91.3%)	29 (55.8%)	≤0.001
Initial nutrition therapy (hours)	48 (12–168)	76 (12–240)	0.208
Initial time of nutrition support			
≤48 h, *n* (%)	18 (39.1%)	17 (32.7%)	0.193
>48 h, *n* (%)	15 (32.6%)	26 (50%)	
Complications, *n* (%)			
Acute kidney injury, *n* (%)	34 (73.9%)	28 (53.8%)	0.004
Upper gastrointestinal bleeding, *n* (%)	2 (4.3%)	4 (7.7%)	0.491
Vasopressors, *n* (%)	27 (58.7%)	23 (44.2%)	0.153

Abbreviations: BUN, blood urea nitrogen; mNUTRIC-Score, modified Nutrition Risk in the Critically Ill-Score; IQR, interquartile range; PaCO_2_, arterial partial carbon dioxide pressure; PaO_2_/FiO_2_ ratio, partial pressure of arterial oxygen to fraction of inspired oxygen.

**Table 3:** Comparison of each item among 28-day survivors and nonsurvivors.

Clinical characteristics	Survivors, *n* = 27	Nonsurvivors, *n* = 71	*p* value
Female *n*, (%)	10 (37%)	21 (29.6%)	0.478
Male *n*, (%)	17 (63%)	50 (70.4%)	
Age, mean	49.51 ± 12.73	60.15 ± 12.91	≤0.001
Comorbidities	1 (0–4)	1 (0–7)	0.101
Charlson comorbidity index	2 (0–4)	3 (0–9)	≤0.001
Severity of disease			
SOFA score	6.22 ± 2.53	8.25 ± 2.56	≤0.001
APACHE II	14.59 ± 7.11	20.76 ± 6.09	≤0.001
Biochemical			
Glucose (mg/dL)	154 (77–533)	164 (45–452)	0.387
Creatinine (mg/dL)	0.83 (0.39–20.23)	1.48 (0.30–13.20)	≤0.001
BUN (mg/dL)	22.60 (10.50–137)	34 (9–108)	≤0.001
Albumin (g/dL)	2.59 ± 0.44	2.54 ± 0.50	0.667
C-reactive protein (mg/dL)	197.37 ± 167.50	199.95 ± 123.78	0.934
Arterial blood gas			
pH	7.35 ± 0.14	7.30 ± 0.14	0.156
PaCO_2_ (mmHg)	38 (14–163)	45 (13–114)	0.694
Bicarbonate (mmol/L)	23.37 ± 7.13	22.50 ± 5.83	0.538
Lactate (mmol/L)	1.80 (0.60–4.30)	2.10 (0.90–7)	0.056
PaO_2_/FiO_2_ (mmHg)	79 (31–277)	74 (41–366)	0.565
Outcomes			
Mechanical ventilation (days)	9 (3–40)	7 (1–27)	0.024
Total mNUTRIC-Score, median (IQR), points	2 (0–7)	5 (0–8)	≤0.001
mNutric-Score level, *n* (%)	4 (14.8%)	42 (59.2%)	≤0.001
Hospital length of stay (days)	17 (10–96)	11 (2–28)	≤0.001
Initial nutrition therapy (hours)	76 (12–240)	72 (24–240)	0.713
Initial time of nutrition therapy			
≤48 h, *n* (%)	11 (40.7%)	24 (33.8%)	0.490
>48 h, *n* (%)	16 (59.3%)	25 (35.2%)	
Complications, *n* (%)			
Acute kidney injury, *n* (%)	10 (37%)	52 (73.2%)	≤0.001
Upper gastrointestinal bleeding, *n* (%)	2 (7.4%)	4 (5.6%)	0.744
Vasopressors, *n* (%)	11 (40.7%)	39 (54.9%)	0.209

Abbreviations: APACHE II, Acute Physiology and Chronic Health Evaluation II; BUN, blood urea nitrogen; mNUTRIC-Score, modified Nutrition Risk in the Critically Ill-Score; IQR, interquartile range; PaCO_2_, arterial partial carbon dioxide pressure; PaO_2_/FiO_2_ ratio, partial pressure of arterial oxygen to fraction of inspired oxygen; SOFA, Sequential Organ Failure Assessment.

**Table 4:** Logistic regression of significant variables for 28-day mortality.

	OR	CI 95%		*p* value
Age	1.062	1.024	1.102	≤0.001
Charlson comorbidity index	1.808	1.294	2.524	≤0.001
SOFA score	1.387	1.133	1.699	0.002
APACHE II	1.179	1.079	1.290	≤0.001
Creatinine (mg/dL)	1.081	0.855	1.367	0.516
BUN (mg/dL)	1.035	1.004	1.066	0.025
Total mNUTRIC-Score, median (IQR), points	1.812	1.358	2.416	≤0.001
mNUTRIC-Score (≥5)	8.328	2.604	26.630	≤0.001
Acute kidney injury	5.943	2.217	15.932	≤0.001

## Data Availability

While the relevant data are included within the article, some data might not be shared as we are concerned about patients' privacy (the database includes names and hospital registry).
